# Patient-Reported Financial Burden Following Stereotactic Body Radiation Therapy for Localized Prostate Cancer

**DOI:** 10.3389/fonc.2022.852844

**Published:** 2022-03-25

**Authors:** Tamir N. Sholklapper, Michael L. Creswell, Alexandra T. Payne, Michael Markel, Abigail Pepin, Michael Carrasquilla, Alan Zwart, Malika Danner, Marilyn Ayoob, Thomas Yung, Brian Collins, Deepak Kumar, Nima Aghdam, Simeng Suy, Ryan A. Hankins, Keith Kowalczyk, Sean P. Collins

**Affiliations:** ^1^ Georgetown University School of Medicine, Washington, DC, United States; ^2^ Department of Radiation Medicine, Georgetown University Hospital, Washington, DC, United States; ^3^ Julius L. Chambers Biomedical Biotechnology Research Institute, North Carolina Central University, Durham, NC, United States; ^4^ Department of Radiation Medicine, Beth Israel Deaconess, Boston, MA, United States; ^5^ Department of Urology, Georgetown University Hospital, Washington, DC, United States

**Keywords:** prostate cancer, SBRT (stereotactic body radiation therapy), CyberKnife, financial toxicity, HRQoL (health-related quality of life)

## Abstract

**Introduction and Objectives:**

In patients with localized prostate cancer, 5-fraction, stereotactic body radiation therapy (SBRT) has been found to offer comparable oncologic outcomes and potential for improved treatment compliance compared to conventional, 40-plus fraction radiation therapy (RT). Recent studies of oncologic patient experiences have highlighted both the impact of therapy-associated financial toxicity (FT) on treatment adherence and health-related quality of life (HRQOL).

**Methods:**

A cross-sectional assessment of FT after SBRT was performed using the 12-item COST questionnaire. The total questionnaire score (range 0–44) was used to evaluate the FT grade (0–3), with a higher COST value representing lower grade. The patient zip code was used to approximate the distance from the index hospital. Univariate and multivariate analyses of the average COST score (0–4) are performed.

**Results:**

The response rate was 57.5% (332 of 575 consented patients) with 90.7%, 8.2%, and 1.1% experiencing grade 0, 1, and 2 FT, respectively, with no grade 3. Unemployment or disability, non-white race, low income, and concurrent hormonal therapy were associated with a statistically significant worse FT (lower COST value) on univariate and multivariate analyses (p < 0.05). Education level and insurance status significant were evaluated on univariate analysis only. There was a non-statistically significant difference in age, marital status, time since treatment, and distance from the index hospital.

**Conclusions:**

SBRT was associated with low FT. However, statistically significant socioeconomic disparities in FT remain despite ultra-hypofractionated treatment.

## Introduction

Financial toxicity (FT) is a patient-centric experience of the financial burden of disease and its management (1–3). FT has historically been analyzed objectively by looking at a patient’s direct cost of disease management. Some studies report that almost half of patients undergoing cancer treatment fully deplete their life assets by 2 years post-diagnosis, with average losses approaching nearly $100,000 by year 4 (4). In the past decade, the understanding of FT has been broadened to include the subjective financial burden and indirect costs (e.g., loss of work for patient or caretaker) associated with disease (1–3).

To date, many of the studies of FT for patients with prostate cancer have relied on non-validated, subjective instruments (5, 6). The Comprehensive Score for Financial Toxicity - Functional Assessment of Chronic Illness Therapy (COST-FACIT) questionnaire (**Supplement 1**), a 12-item validated instrument for assessing financial toxicity (FT), was initially validated in patients with advanced cancer; it has recently been validated in the radiation oncology setting (7, 8). This work was recently expanded in the surgical management of prostate cancer (9).

Of all urologic malignancies, most financial toxicity research has focused on prostate cancer (1). In the past decade, emerging studies have begun identifying an association between FT for patients with prostate cancer and clinically significant factors such as health-related quality of life (HRQoL), compliance, and even survival (1, 7, 9–11). However, not all treatments are equivalent in FT, and radiation therapy (RT) is generally thought to be associated with a more severe FT than radical prostatectomy or active surveillance (5, 12).

Given the significant financial distress faced by cancer patients and the potential association between FT and other clinically significant outcomes, we must strive for highly effective treatment options that minimize FT. In patients with localized prostate cancer, 5-fraction stereotactic body radiation therapy (SBRT), moderately hypofractionated radiotherapy, and permanent seed implants offer comparable oncologic outcomes and potential for improved treatment compliance compared to conventional, 40-plus fraction radiation therapy (RT) (5, 13–17). Unfortunately, to date, there is scant data reporting FT in patients who receive prostate SBRT.

In this study, we use the COST-FACIT to evaluate the patient-reported financial toxicity after SBRT for localized prostate cancer. We aim to evaluate patient and treatment factors associated with worse financial toxicity.

## Materials and Methods

### Study Cohort

Patients eligible for this cross-sectional study had histologically confirmed, localized prostate cancer and were treated at MedStar Georgetown University Hospital with five fractions SBRT. From 2012 to 2020, a total of 575 patients consented to participate in this IRB-approved (IRB 12-1175) prospective institutional quality-of-life study. Surveys were mailed to all participants or, if applicable, collected at an in-person treatment or posttreatment visit.

### Outcomes

The primary outcome of this study was financial toxicity as assessed by the 12-item COST-FACIT questionnaire (version 2, www.facit.org/measures/FACIT-COST). After considering items with reverse values, the COST score was calculated as an average of the 11 scored items (range 0–4). In accordance with the FACIT-scoring guidelines, only surveys with at least 80% of the scored questions completed (at least 9 of 11) were included. A lower COST score indicated more severe financial toxicity.

COST grade (range 0–3) was determined by the total COST sum (range 0–44), which was the score calculated using the questionnaire. As described by D’Rummo et al., the COST sum was further broken down into COST sum categories of “≥26,” “14–25,” “1–13,” and “0” representing COST grades 0, 1, 2, and 3, respectively (8). Only surveys with 100% of the scored questions completed were included. A higher COST grade indicated more severe financial toxicity.

### Exposure

SBRT treatment planning and delivery were conducted as previously described (18). Briefly, gold fiducials were placed into the prostate. Fused CT and MR images were used for treatment planning. The clinical target volume (CTV) included the prostate and proximal seminal vesicles. The planning target volume (PTV) equaled the CTV expanded 3 mm posteriorly and 5 mm in all other dimensions. The prescription dose was 35–36.25 Gy to the PTV delivered in five fractions of 7–7.25 Gy over 1 to 2 weeks.

### Covariates

Surveys included questions related to patient age, marital status, employment status, level of education, race and ethnicity, income level, health insurance, and hormonal therapy. Distance from the index hospital was determined using the patient-reported zip code converted to approximate latitude and longitude. The Haversine formula was used to determine the shortest distance between each set of coordinates. Time since treatment was calculated as a difference in months between survey date and treatment day 1.

### Statistical Analysis

Baseline patient characteristics were summarized by the number of patients and percentage of respondents by variable. These characteristics were further delineated by COST grade, and differences among categorical survey responses were evaluated using Fisher’s exact test and the one-way analysis of variance (ANOVA) method for continuous age variables. Differences in average COST score were presented by mean, difference from population mean, and range. A visual representation of the COST score was performed using a violin density plot by categorical response.

Univariate analysis and multivariate analysis were used to determine factors associated with the average COST score. For analysis, race was dichotomized as white and other, and time since SBRT was dichotomized as ≤ or >6 months. Univariate analysis of age was performed using linear regression and Wilcoxon rank-sum test or Kruskal–Wallis one-way analysis of variance for the remainder of ordinal or nominal covariates. The multivariate model was performed *via* multiple regression using the method of least squares. Backward selection was used to select variables for the multivariate model until only significant variables with *p* < 0.05 remained. All tests were two-tailed, and a *p* value <0.05 was considered significant. JMP^®^ Pro, version 15.0.0 (SAS Institute Inc., Cary, NC, 1989–2021), was used to perform the statistical analyses.

## Results

The questionnaire response rate was 57.5%, with 332 of 575 patients completing the questionnaire and included in the analysis. Demographics and adjunct hormonal therapy are reported in **Table 1**. The median age of the cohort was 76, with a range of 54 to 92 years. A majority of the population were married (n = 257; 77.7%), retired (232; 71.4%), graduate degree-holding (207; 65.1%), white (264; 80.2%), with an income ≥$100,000 (214; 69.5%), and living within 25 miles of the hospital (247; 74.8%). Nearly the entire cohort reported having a health insurance (323; 98.2%). Of the respondents, most were more than 6 months past treatment (305; 91.9%).

**Table 1:** Demographics and baseline characteristics by COST toxicity grade.

	Overall		COST grade						
			Grade 0		Grade 1		Grade 2		
	N	(%)	N	(%)	N	(%)	N	(%)	*p* -value
Age at survey									0.2421
Treatment median, Y (range)	70	(47–90)							
Median, Y (range)	76	(54–92)							
<50	0	(0.0%)	0	(0.0%)	0	(0.0%)	0	(0.0%)	
51–64	26	(7.8%)	17	(6.7%)	4	(17.4%)	1	(33.3%)	
65–75	134	(40.4%)	109	(42.7%)	12	(52.2%)	0	(0.0%)	
>75	172	(51.8%)	129	(50.6%)	7	(30.4%)	2	(66.7%)	
Marital status									0.3433
Single	30	(9.1%)	20	(7.9%)	3	(13.0%)	1	(33.3%)	
Married	257	(77.6%)	197	(77.6%)	16	(69.6%)	2	(66.7%)	
Widowed	22	(6.6%)	19	(7.5%)	2	(8.7%)	0	(0.0%)	
Divorced	14	(4.2%)	11	(4.3%)	1	(4.3%)	0	(0.0%)	
Long-term partner	8	(2.4%)	7	(2.8%)	1	(4.3%)	0	(0.0%)	
Employment status									**0.0045**
Working	87	(26.8%)	71	(28.3%)	7	(30.4%)	1	(33.3%)	
Retired	232	(71.4%)	179	(71.3%)	14	(60.9%)	1	(33.3%)	
Disabled	3	(0.9%)	0	(0.0%)	1	(4.3%)	1	(33.3%)	
Unemployed	3	(0.9%)	1	(0.4%)	1	(4.3%)	0	(0.0%)	
Education									0.0872
No HS diploma	2	(0.6%)	0	(0.0%)	0	(0.0%)	0	(0.0%)	
HS/GED	22	(6.7%)	14	(5.6%)	3	(13.0%)	1	(33.3%)	
College	97	(29.6%)	74	(29.4%)	7	(30.4%)	1	(33.3%)	
Graduate or professional	207	(63.1%)	164	(65.1%)	13	(56.5%)	1	(33.3%)	
Race									**0.0460**
White or Caucasian	264	(80.2%)	208	(81.9%)	15	(65.2%)	2	(66.7%)	
Black or AA	51	(15.5%)	34	(13.4%)	7	(30.4%)	0	(0.0%)	
Latino or Hispanic	4	(1.2%)	3	(1.2%)	1	(4.3%)	0	(0.0%)	
Asian	8	(2.4%)	7	(2.8%)	0	(0.0%)	1	(33.3%)	
Other	2	(0.6%)	2	(0.8%)	0	(0.0%)	0	(0.0%)	
Income									**<.0001**
$0–14,999	4	(1.3%)	1	(0.4%)	0	(0.0%)	1	(33.3%)	
$15,000–49,999	25	(8.1%)	15	(6.2%)	2	(8.7%)	1	(33.3%)	
$50,000–99,000	65	(21.1%)	45	(18.6%)	11	(47.8%)	0	(0.0%)	
$100,000–149,999	77	(25.0%)	62	(25.6%)	6	(26.1%)	1	(33.3%)	
$150,000 or more	137	(44.5%)	119	(49.2%)	4	(17.4%)	0	(0.0%)	
Distance from hospital									0.3180
0–25 miles	247	(74.8%)	191	(75.5%)	15	(65.2%)	3	(100.0%)	
26–50 miles	29	(8.8%)	19	(7.5%)	3	(13.0%)	0	(0.0%)	
51–100 miles	18	(5.5%)	10	(4.0%)	3	(13.0%)	0	(0.0%)	
101–300 miles	12	(3.6%)	12	(4.7%)	0	(0.0%)	0	(0.0%)	
>300 miles	24	(7.3%)	21	(8.3%)	2	(8.7%)	0	(0.0%)	
Health insurance									**0.0481**
No	6	(1.8%)	1	(0.4%)	1	(4.5%)	0	(0.0%)	
Yes	323	(98.2%)	252	(99.6%)	21	(95.5%)	3	(100.0%)	
Time since SBRT									0.2549
<6 months	27	(8.1%)	21	(8.2%)	1	(4.3%)	1	(33.3%)	
>6 months	305	(91.9%)	234	(91.8%)	22	(95.7%)	2	(66.7%)	
Hormonal therapy									0.2627
None	248	(75.6%)	195	(77.1%)	12	(54.5%)	2	(66.7%)	
Previously	61	(18.6%)	47	(18.6%)	5	(22.7%)	1	(33.3%)	
Currently	19	(5.8%)	11	(4.3%)	5	(22.7%)	0	(0.0%)	

p-value for age derived via one-way ANOVA, with the remainder calculated via Fisher’s exact test.

Bolded values represent statistical significance with p-values < 0.05.

**Figure 1:**
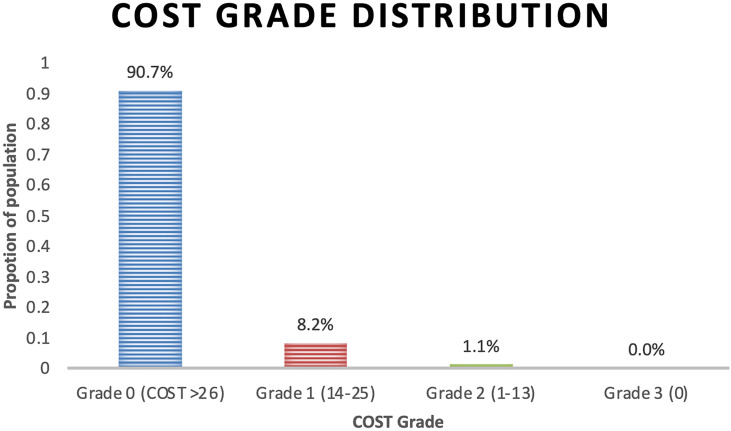
COST Grade toxicity distribution by proportion of population.

The COST grade breakdown for the population was 90.7%, 8.2%, and 1.1% for grades 0, 1, and 2, respectively, with no grade 3 toxicity (**Figure 1**). Employment status (*p* = 0.0045), race (*p* = 0.0481), and health insurance status (*p* = 0.0481) significantly differed by COST grade groupings. Patient characteristics and COST grade grouping demonstrated no statistically significant differences in education level, distance from hospital, time since treatment, and hormonal therapy. Similarly, analysis of age revealed a non-statistically significant difference in age at treatment among COST grade groupings.

**Figure 2:**
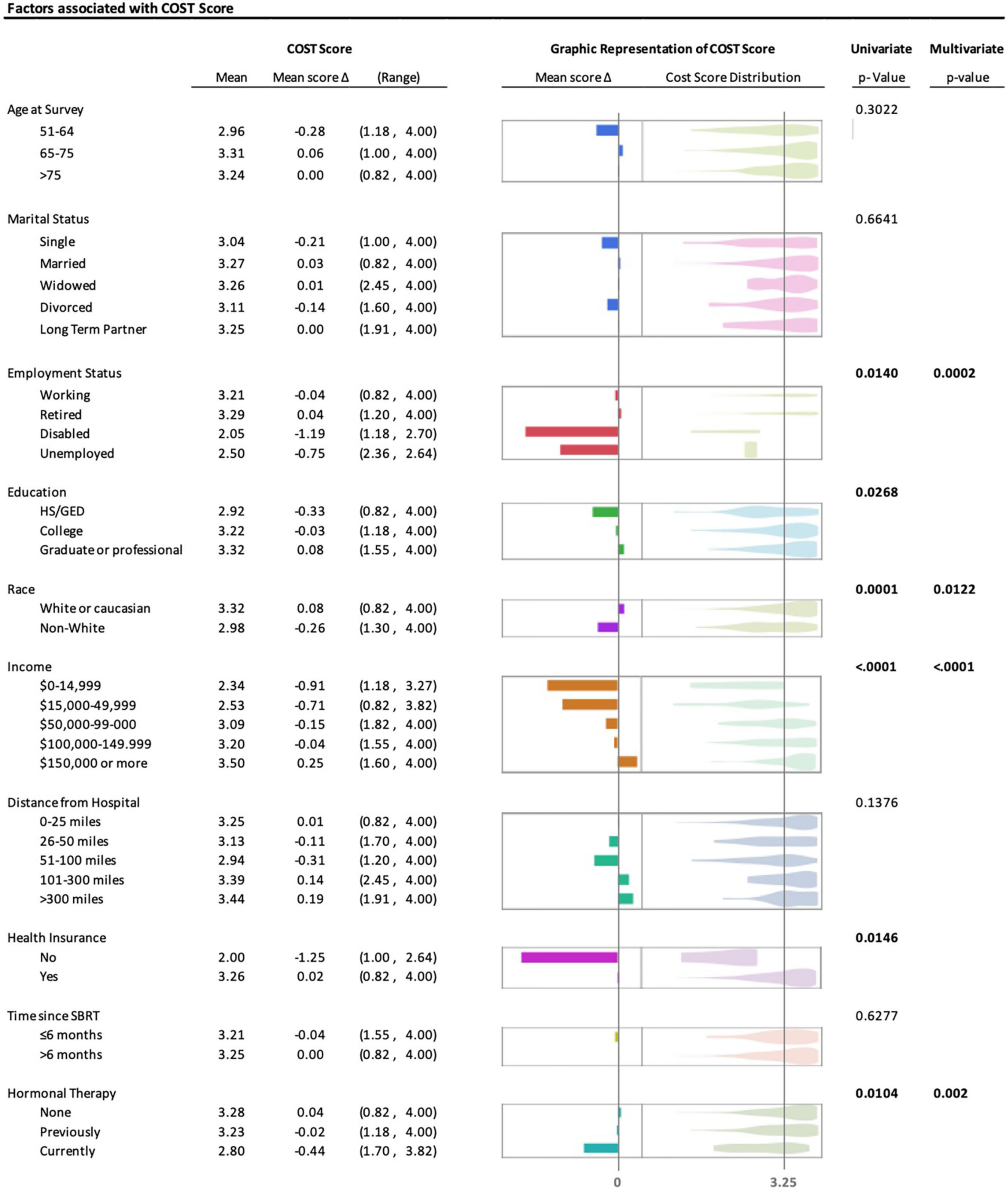
UVA and MVA of covariates associated with greater financial toxicity (COST Score). Mean COST Score, difference from population mean, and range in the first column; graphic representation of difference in mean COST Score and violin plot showing the distribution of individual COST Score values; vertical line represents the population mean COST Score. *p*-value of univariate analysis using linear regression for age, and Wilcoxon rank-sum or Kruskal–Wallis test for remainder of variables; multivariate analysis using multiple regression with standard least-square method.

The average COST score for the cohort was 3.25 out of 4. In **Figure 2**, univariate analysis of covariates associated with COST score was significant for employment status (mean score: retired 3.29, working 3.21, disabled 2.05, unemployed 2.50; *p* = 0.0140), education (high-school or GED 2.92, college 3.22, graduate or professional 3.32; *p* = 0.0268), race (white or Caucasian 3.32 versus non-white 2.98; *p* = 0.0001), income (<$15,000 2.34, ≥$150,000 3.50; *p* < 0.0001), health insurance (no health insurance 2.00 versus with health insurance 3.26; *p* = 0.0146), and hormonal therapy (current 2.80, previous 3.23, never 3.28; *p* = 0.0104). There was no difference in COST score by age, marital status, distance from the hospital, or time since treatment. Employment status (*p* = 0.0002), race (*p* = 0.0122), income (*p* < 0.0001), and hormonal therapy (*p* = 0.0020) remained significant on multiple regression.

## Discussion

Financial toxicity can have a significant impact on the livelihood of patients and their support system. This is especially evident given that, for patients over 50 years old, approximately 42% will completely deplete their assets within 2 years of a new cancer diagnosis (4). Further, subjective FT may have a greater negative impact on HRQoL than objective FT (10). The present study is the first to use COST-FACIT to evaluate patient-reported FT in patients with new prostate cancer diagnoses receiving RT as primary therapy.

The first publication of COST-FACIT was in 2017 and evaluated FT in patients with stage IV malignancies as part of the validation of the scoring tool (7). Since then, COST-FACIT has been used to evaluate FT in the radiation oncology setting. In a study by D’Rummo et al., 167 patients with a variety of primary malignancies and treatment courses were evaluated using this metric. Of these, 56.3% of patients experienced grade 1 FT. In our study, over 90% of patients experienced grade 0 FT (8). The reason for such low FT is likely multifactorial and may relate to the overwhelming proportion of men who were retired and report high-income levels, as well as the 5-treatment course of SBRT. Interestingly, we found no significant difference in FT for patients who were within 6 months of SBRT. In the study by D’Rummo et al., they found that patients who were within 6 months of RT were more likely to experience FT (8).

In terms of factors associated with worse FT in our patient population, unemployment or disability, non-white race, low income, and concurrent hormonal therapy were associated with a statistically significant worse FT on univariate and multivariate analyses. In the study by Stone et al., patients with localized prostate cancer who identified as either Black or Hispanic had a higher odds of financial burden when adjusted for age, insurance, education, marriage, comorbidities, and D’Amico risk group (5). However, in the present study, racial differences did not account for the greatest difference in FT. In order of decreasing severity, patients who did not have health insurance, who were disabled, or who had an income less than $14,999 annually were the three groups reporting the worst FT. A recently published abstract by Gorovets et al. reported using COST-FACIT to evaluate FT in RT. In this abstract, Gorovets et al. evaluated FT in 373 men who received SBRT, moderately hypofractionated radiotherapy, brachytherapy, or combination EBRT/brachytherapy (19). Overall, the authors report low levels of FT for each modality and SBRT had the lowest FT. Despite this, 5%–10% of patients report high levels of distress related to treatment costs. However, these patients were primarily white, married, insured, and with high annual incomes, all of which are protective factors for FT (19).

In a study by Gilligan et al. looking at objective financial burden in patients with newly diagnosed malignancies, the authors similarly found that low income is associated with a greater burden. However, they also found that patients who were retired had a higher odds of depleting their assets (4). They also suggest that improved oncologic prognosis lends itself to higher risk of asset depletion (4). Given the chronicity of most prostate cancer diagnoses, it is too early to tell if the same is true of FT in the urologic patient population.

Previous studies have also investigated the direct and indirect objective financial burden of patients with prostate cancer. These data are generated by calculating actual costs to patients rather than a validated survey such as COST-FACIT. Jayadevappa et al. reported the direct and indirect costs across time to men with prostate cancer who were treated by radical prostatectomy (RP) or EBRT. At 3 months, the total cost to patients was $2010 *vs*. $5576 for EBRT and RP, respectively. However, this effect reversed at 6 months (sum costs; EBRT: $2133, RP:$1776) and at 2 years (sum costs; EBRT: $871, RP: $458) (20). An important caveat to this study is that all men had health insurance. It is likely that the objective financial burden to under- and uninsured patients treated for prostate cancer may be greater. Additionally, practice patterns have changed with the introduction of SBRT as an alternative treatment to EBRT.

Understanding subjective FT can be as valuable as understanding objective FT and highlights one of the strengths of the present study. In a study of FT experienced by patients with urologic malignancies by Ting et al., increasing subjective FT had a greater negative impact on HRQoL than objective FT (10). The authors defined objective FT as healthcare cost-to-income ratio greater than 0.4 and subjective FT and HRQoL using the validated Personal Financial Well-being Scale and Functional Assessment of Cancer Therapy – General 7 Items Scale, respectively. While, notably, their study was based out of Malaysia, a middle-income country with a universal healthcare system, they demonstrated that universal health coverage does not eliminate the burden experienced by patients. While the present study did not capture objective FT beyond patient-reported income, the overwhelming majority of patients reported some form of healthcare coverage, therefore making it possible to delineate additional socioeconomic factors associated with greater FT.

Our study should be considered in the context of its limitations. This is a cross-sectional representation of a prospective study with a majority of respondents beyond 6 months posttreatment with SBRT. In similar studies, financial toxicity appears to be front-loaded; it is therefore possible that the distribution financial toxicity of men with prostate cancer treated by SBRT may be shifted in our cohort (5, 20). Future expansion of this cross-sectional study will capture longitudinal changes in financial toxicity as it relates to time since treatment as well as pretreatment baseline. Another limitation is the population of survey respondents, which is 80.2% white. In one of our prior publications, the 10-year demographics of our institutional prostate-cancer population, 46% of the population is white, 48% black, and 6% other (15). This sampling limitation may have also impacted the number of retired patients, graduate degree-holding, and reporting high annual incomes; however, these data were not previously evaluated in our patient population. Lastly, being the first study on FT in patients who have had SBRT for prostate cancer is both a strength and limitation, and due to the relatively small number of patients, non-parametric statistical tests were used for this analysis. Consenting additional patients and prospective financial toxicity collection should enable us to better account for many of these limitations in future analyses.

As mentioned, future financial toxicity research in patients with prostate cancer should involve longitudinal analyses. The integration of HRQoL and disease metrics will enhance the long-term analysis of FT. Several comparator studies of financial toxicity will also better elucidate the impact of treatment choice on patients with prostate cancer, specifically comparison of SBRT to active surveillance, prostatectomy, and systemic therapies.

## Conclusion

Understanding the aspects of oncologic care that directly impact patient experience, treatment adherence, and HRQOL is of utmost importance. SBRT is associated with low overall FT. However, statistically significant socioeconomic disparities in FT remain despite ultra-hypofractionated treatment. Patients who are unemployed or have a disability, non-white, low income, or on hormonal therapy are more likely to experience significant FT after SBRT for prostate cancer.

## Data Availability Statement

The datasets presented in this article are not readily available because as per the wishes of the patients, the patient dataset is only available to those conducting research at the Georgetown University Medical Center. Requests to access the datasets should be directed to Sean Collins, SPC9@gunet.georgetown.edu.

## Ethics Statement

The studies involving human participants were reviewed and approved by the Georgetown University Institutional Review Board. The patients/participants provided their written informed consent to participate in this study. The patients/participants provided their written informed consent to participate in this study.

## Author Contributions

TS, MC, AP, and SC contributed to the conception and design of the study. AZ distributed the questionnaires and organized the database. TS, MC, and SC wrote the first draft of the manuscript. All authors contributed to the manuscript revision and read and approved the submitted version.

## Funding

The Department of Radiation Medicine at Georgetown University Hospital receives a grant from Accuray to support a research coordinator. We gratefully acknowledge the grant R01MD012767 from the National Institute on Minority Health and Health Disparities (NIMHD), NIH, to SC. This work was supported by The James and Theodore Pedas Family Foundation.

## Conflict of Interest

SC and BC serve as clinical consultants at Accuray Inc.

The authors declare that the research was conducted in the absence of any commercial or financial relationships that could be construed as a potential conflict of interest.

## Publisher’s Note

All claims expressed in this article are solely those of the authors and do not necessarily represent those of their affiliated organizations, or those of the publisher, the editors and the reviewers. Any product that may be evaluated in this article, or claim that may be made by its manufacturer, is not guaranteed or endorsed by the publisher.

## Abbreviations

ANOVA, analysis of variance; COST-FACIT, Comprehensive Score for Financial Toxicity - Functional Assessment of Chronic Illness Therapy; CTV, clinical target volume; EBRT, external beam radiation therapy; FT, financial toxicity; HRQOL, health-related quality of life; PTV, planning target volume; RT, radiation therapy; RP, radical prostatectomy; SBRT, stereotactic body radiation therapy.
