# Supplementary material for: Patient-Reported Financial Burden Following Stereotactic Body Radiation Therapy for Localized Prostate Cancer
Source: Front Oncol. 2022 Mar 25;12:852844. doi: 10.3389/fonc.2022.852844 (PMC8990911; doi:10.3389/fonc.2022.852844)
Supplement: Supplementary file 1 [file DataSheet_1.pdf]

COST – FACIT (Version 2)

Below is a list of statements that other people with your illness have said are important**. Please circle or mark one number per line to indicate your response as it applies to the <u>past 7 days</u>.**

		Not at all	A little bit	Some-what	Quite a bit	Very much
FT1	I know that I have enough money in savings, retirement, or assets to cover the costs of my treatment	0	1	2	3	4
FT2	My out-of-pocket medical expenses are more than I thought they would be	0	1	2	3	4
FT3	I worry about the financial problems I will have in the future as a result of my illness or treatment	0	1	2	3	4
FT4	I feel I have no choice about the amount of money I spend on care	0	1	2	3	4
FT5	I am frustrated that I cannot work or contribute as much as I usually do	0	1	2	3	4
FT6	I am satisfied with my current financial situation	0	1	2	3	4
FT7	I am able to meet my monthly expenses	0	1	2	3	4
FT8	I feel financially stressed	0	1	2	3	4
FT9	I am concerned about keeping my job and income, including work at home	0	1	2	3	4
FT10	My cancer or treatment has reduced my satisfaction with my present financial situation	0	1	2	3	4
FT11	I feel in control of my financial situation	0	1	2	3	4
FT12	My illness has been a financial hardship to my family and me	0	1	2	3	4

Copyright 2014, FACIT and The University of Chicago
